# The Role of Neutrophil Extracellular Traps (NETosis) in Systemic Lupus Erythematosus: Molecular Mechanisms, Pathogenic Roles, Diagnostic Biomarkers, and Emerging Therapeutic Strategies

**DOI:** 10.3390/ijms27198530

**Published:** 2026-09-24

**Authors:** Gokul Patil

**Affiliations:** Department of Internal Medicine, University of Iowa Health Care, Iowa City, IA 52242, USA; gokul-patil@uiowa.edu; Tel.: +1-319-335-7712

**Keywords:** neutrophil extracellular traps, NETosis, systemic lupus erythematosus, low-density granulocytes, PAD4, DNase1, type I interferon, biomarkers, therapeutic targets, lupus nephritis

## Abstract

Neutrophil extracellular traps (NETs) are web-like structures comprising chromatin scaffolds decorated with cytotoxic granule proteins released during a specialized form of neutrophil cell death termed NETosis. Although NETs function as a critical antimicrobial defense mechanism, their dysregulation contributes significantly to the pathogenesis of systemic lupus erythematosus (SLE). In SLE patients, both excessive NET formation by proinflammatory low-density granulocytes (LDGs) and impaired NET clearance due to reduced or acquired DNase1/DNase1L3 activity create a pathogenic cycle that amplifies autoimmune activation. NETs serve as both a source of autoantigens and an immunological amplifier, driving Type I interferon responses, complement activation, B cell autoimmunity, endothelial dysfunction, and thrombosis. Recent advances have identified circulating biomarkers of NETosis, including cell-free DNA (cfDNA), myeloperoxidase-DNA complexes, and citrullinated histone H3, that correlate with SLE disease activity and organ involvement. Emerging therapeutic strategies targeting NETosis pathways, including peptidylarginine deiminase 4 (PAD4) inhibitors, recombinant DNase therapy, and complement modulators, show promise across preclinical studies and selected early clinical investigations. This comprehensive review synthesizes current mechanistic understanding of NET biology in SLE, examines the complex interplay between NETosis and innate/adaptive immunity, evaluates clinical implications for diagnosis and prognosis, and critically assesses translational opportunities and remaining challenges in developing NETosis-targeted interventions for this devastating autoimmune disease.

## 1. Introduction

Systemic lupus erythematosus represents a prototypical autoimmune disease characterized by widespread immune dysregulation, generation of pathogenic autoantibodies targeting nuclear antigens, and multi-organ inflammation culminating in significant morbidity and mortality [1]. Historically, SLE was conceptualized primarily as a disorder of adaptive immunity, with particular emphasis on B cell hyperactivation and autoantibody production. However, accumulating evidence over the past decade has fundamentally reshaped our understanding of SLE pathogenesis, positioning innate immune dysfunction, particularly defects in neutrophil function and fate, as a central mechanistic driver of disease initiation and progression [2].

Among the most significant discoveries reshaping lupus immunobiology is the recognition that neutrophil extracellular traps (NETs) and the process of their formation (NETosis) constitute a critical pathogenic mechanism in SLE [3]. NETs are distinctive, web-like extracellular structures composed of decondensed chromatin decorated with histones, neutrophil elastase, myeloperoxidase, and numerous antimicrobial proteins [4]. While NETs normally serve as antimicrobial scaffolds that trap and kill diverse pathogens, in SLE the balance between NET formation and clearance becomes critically dysregulated [5]. This imbalance manifests as excessive NET formation coupled with impaired NET degradation, creating a vicious cycle in which NETs act simultaneously as autoantigen reservoirs, activators of pattern recognition receptors, and amplifiers of Type I interferon responses, processes that perpetuate and accelerate autoimmunity [6].

Although this review focuses on SLE, dysregulated NET formation and persistence are not unique to lupus. Pathogenic roles for NETs have also been described in rheumatoid arthritis, systemic sclerosis, inflammatory bowel disease, antiphospholipid syndrome, and other immune-mediated inflammatory disorders, where NET-derived nucleic acids, modified proteins, proteases, and oxidants can amplify autoantigen exposure, innate immune signaling, vascular injury, and tissue inflammation [7]. SLE nevertheless provides a particularly instructive model because excessive NET formation, defective extracellular DNA clearance, type I interferon signaling, autoantibody production, complement activation, and thromboinflammation converge within the same disease.

The pathogenic intersection of NETosis and SLE operates through multiple converging mechanisms. A distinct population of circulating neutrophils termed low-density granulocytes (LDGs), preferentially enriched in SLE patients, demonstrates heightened spontaneous NET formation capacity and produces elevated levels of Type I interferons [8]. Simultaneously, genetic and acquired defects in DNase1 and DNase1L3 activity compromise NET clearance, allowing circulating NETs to persist and accumulate, forming pathogenic immune complexes that deposit in renal glomeruli, trigger complement activation, and perpetuate inflammation [9]. These accumulating NETs expose immunogenic autoantigens, activate endosomal Toll-like receptors (TLR7/9), and engage the cytosolic cGAS-STING pathway, collectively amplifying Type I interferon signaling and enabling breakdown of self-tolerance [10].

The clinical relevance of NETosis in SLE is underscored by emerging biomarker data demonstrating that circulating NET components, including cell-free DNA, myeloperoxidase-DNA complexes, and citrullinated histone H3, correlate strongly with disease activity, organ involvement, and treatment response [11]. These discoveries have catalyzed development of novel therapeutic approaches targeting NETs, ranging from PAD4 inhibitors that prevent histone citrullination and NET formation, to recombinant DNase therapies that degrade existing NETs, to complement inhibitors that reduce NET-induced organ damage. Early clinical experience with several of these interventions suggests therapeutic promise, although translation remains incomplete due to requirements for patient stratification, determination of optimal treatment windows, and careful preservation of antimicrobial host defenses.

This comprehensive review synthesizes current understanding of NETosis mechanisms in SLE, examines molecular pathways linking aberrant NET formation to lupus pathogenesis, evaluates diagnostic and prognostic applications of NET-derived biomarkers, and critically assesses emerging therapeutic strategies. By integrating findings from basic science, animal models, and human clinical studies, this review identifies knowledge gaps and priorities for future investigation, with particular attention to translational pathways most likely to improve outcomes in this complex and protean disease.

## 2. Biology of NETosis: Historical Context and Definition

### 2.1. Historical Discovery and Paradigm Shifts

The discovery of NETosis represents a paradigm shift in our understanding of neutrophil biology and cell death pathways. Brinkmann and colleagues’ seminal 2004 observation that neutrophils could extrude their nuclear material as web-like DNA structures decorated with antimicrobial proteins challenged the long-standing dogma that neutrophils possessed only two fates: phagocytosis-based killing followed by apoptosis or necrosis [12]. This discovery identified NETosis as a distinct form of programmed cell death fundamentally different from classical apoptosis, with its own regulatory mechanisms and functional consequences. Initial conceptualizations emphasized the antimicrobial utility of NETs, with emphasis on their capacity to immobilize and kill diverse bacterial, fungal, and parasitic pathogens in a manner independent of classical phagocytosis [12].

However, subsequent investigations revealed the dual-edged nature of NETs. While NETs clearly contribute to innate antimicrobial defense, excessive or dysregulated NETosis produces injurious consequences, including inflammatory tissue injury, autoimmune activation, thrombosis, and propagation of chronic inflammation [4]. The recognition that NETosis drives autoimmune pathology emerged principally from studies of systemic lupus erythematosus, where aberrant NET formation and impaired NET clearance converge to produce a chronic, self-perpetuating inflammatory state [4]. This shift toward understanding NETosis as a context-dependent process, beneficial in acute infections but pathogenic when dysregulated, has reshaped the landscape of NET-related research, particularly in autoimmune diseases [4].

### 2.2. NET Composition and Molecular Architecture

NETs are morphologically distinctive structures comprising a DNA scaffold derived from decondensed chromatin that becomes studded with nuclear and cytoplasmic proteins [12]. Individual NET chromatin fibers are typically on the nanometer scale in diameter and extend over distances of 100 μm or greater, providing an extensive molecular mesh capable of trapping and sequestering particulate and soluble antigens [7]. This DNA scaffold is composed of both euchromatic and heterochromatic regions, with histone proteins retained in their modified chromatin configuration.

The protein composition of NETs reflects contributions from multiple cellular compartments. Nuclear histones, including H1, H2A, H2B, H3, and H4, become modified during NET formation, particularly through citrullination of arginine residues on histone H3 and H4 by peptidylarginine deiminase 4 (PAD4) [13]. These citrullinated and extracellular histones can function as damage-associated molecular patterns by pattern recognition receptors and contribute significantly to the immunogenicity of NETs in the context of autoimmunity [14]. Beyond histones, NETs contain diverse neutrophil granule proteins, including azurophilic granule contents (neutrophil elastase, cathepsin G, myeloperoxidase) and specific granule proteins (lactoferrin, gelatinase, NADPH oxidase components) [12]. Antimicrobial peptides including LL37 (cathelicidin), defensins, and other cationic proteins distribute throughout the NET structure and contribute to both antimicrobial and immunostimulatory properties.

Mitochondrial components also contribute to pathogenic NETs, particularly in the context of autoimmunity. Oxidized mitochondrial DNA (mtDNA) represents a particularly potent immunogen that activates both TLR9 and the cytosolic cGAS-STING pathway, driving exaggerated Type I interferon responses in SLE [6]. Recent evidence indicates that mitochondria are actively recruited into developing NETs and that the content of mtDNA in NETs varies substantially depending on the stimulus and cellular context, with particular enrichment in NETs released by low-density granulocytes in SLE patients [5].

## 3. Mechanisms Regulating NET Formation

### 3.1. Suicidal NETosis: NADPH Oxidase-Dependent Pathway

The canonical mechanism of NETosis, termed “suicidal” NETosis, involves a stepwise sequence of biochemical events culminating in controlled nuclear dissolution and extracellular DNA release [6]. This pathway is typically triggered by inflammatory stimuli including bacterial lipopolysaccharide (LPS), complement component C5a, inflammatory cytokines (TNF-α, IL-8), or immune complexes [4]. Engagement of pattern recognition receptors or complement receptors by these triggers activates phospholipase C and other signaling cascades that culminate in generation of reactive oxygen species (ROS), primarily through activation of the NADPH oxidase complex [5].

NADPH oxidase-derived superoxide anion production is essential for suicidal NETosis, as evidenced by the observation that mice genetically deficient in NADPH oxidase components fail to undergo NETosis in response to most stimuli [13]. The generated ROS activate multiple downstream signaling cascades, including calcium influx, mitogen-activated protein kinase (MAPK) activation, and most critically, nuclear localization and activation of serine proteases including neutrophil elastase and cathepsin G [4]. These serine proteases translocate from azurophilic granules into the nucleus, where they cleave nuclear lamins and histones, resulting in partial chromatin loosening and increased chromatin accessibility.

Concomitantly, NADPH oxidase-derived ROS activate peptidylarginine deiminase 4 (PAD4), a calcium-dependent enzyme that catalyzes conversion of arginine residues to citrulline on histone proteins [13]. Histone citrullination reduces the positive charge on histones, weakening histone-DNA interactions and promoting chromatin decondensation. In combination, neutrophil elastase-mediated histone proteolysis and PAD4-mediated histone citrullination produce progressive nuclear envelope breakdown, mixing of nuclear and granular contents, and ultimately membrane rupture with extrusion of the condensed DNA-protein mass characteristic of mature NETs [14].

Importantly, PAD4 is not an obligatory component of every pathway that produces extracellular chromatin structures. PAD4 dependence varies with the stimulus and experimental system, and rapid or vital NET release can occur with limited histone citrullination. Consistent with this heterogeneity, genetic deletion of *Padi4* did not ameliorate nephritis, loss of tolerance, or immune activation in the MRL.Faslpr lupus model, whereas other lupus models have shown benefit from PAD4 loss or inhibition [15]. These divergent findings caution against treating citrullinated histones as a universal surrogate for NET formation and suggest that PAD4-directed therapy will likely affect only selected NET-forming programs.

### 3.2. Vital NETosis: ROS-Independent Rapid Release

An alternative mechanism of NET release, termed “vital” NETosis, operates independently of NADPH oxidase-derived ROS and produces rapid NET extrusion while maintaining cell membrane integrity and allowing retained cytoplasm to support continued cellular survival [6]. This pathway is triggered by certain stimuli including granulysin, fungal products, and particular viral antigens, and operates through rapid calcium influx without requiring NADPH oxidase activation [4]. In vital NETosis, rapid nuclear envelope breakdown permits extrusion of nuclear material without extensive chromatin decondensation, resulting in the release of more condensed DNA structures that retain nucleosome architecture.

Importantly, vital NETosis proceeds without requiring serine protease activation or extensive histone citrullination, leading to NETs with reduced citrullination content compared to suicidal NETosis [14]. The biological and immunological consequences of vital versus suicidal NETosis may differ substantially, with vital NETs potentially exhibiting reduced citrullination-dependent immunogenicity while maintaining intact antimicrobial function. In SLE patients, the relative contribution of vital versus suicidal NETosis to the pathogenic NET burden remains incompletely characterized, although evidence suggests that suicidal NETosis predominates in response to lupus-associated stimuli including immune complexes and Type I interferons. The major pathways of NET formation are summarized in Figure 1.

### 3.3. NETosis Mimics: Distinguishing True NET Formation from Morphologically Similar Processes

Recent critical re-examination of the NETosis literature has identified two distinct biological processes that share morphological features with classical NETosis (extracellular DNA release and chromatin decondensation) but operate through fundamentally different mechanisms and exhibit distinct biological properties [13]. Appreciation of these distinctions is important for accurate understanding of NETosis in SLE and for rational design of therapeutic interventions.

Leukotoxic hypercitrullination (LTH) represents a bacterial immune evasion strategy in which pore-forming bacterial toxins trigger massive elevation of intracellular calcium, leading to hyperactivation of PAD enzymes and excessive histone citrullination culminating in neutrophil death and DNA release [13]. However, LTH differs fundamentally from true NETosis by being NADPH oxidase-independent and lacking antimicrobial effector function; rather than killing bacteria, LTH represents a bacterial strategy to escape immune surveillance. In rheumatoid arthritis, evidence suggests that LTH rather than true NETosis drives generation of citrullinated autoantigens; in SLE, the relative contributions of LTH versus true NETosis remain to be fully elucidated.

Defective mitophagy represents a second NETosis mimic, whereby constitutive defects in selective autophagy of mitochondria (mitophagy) lead to accumulation of dysfunctional mitochondria and mitochondrial DNA in the cytoplasm, particularly in response to interferon-α and immune complexes [13]. When these dysfunctional mitochondria are expelled from cells (a process distinct from true NETosis), they release oxidized mitochondrial DNA that activates the cGAS-STING pathway and drives Type I interferon responses. This process generates highly immunogenic circulating oxidized mtDNA and has historically been misattributed to NETosis; in SLE, this defective mitophagy pathway may contribute substantially to the interferon signature and should be distinguished from true NADPH oxidase-dependent NETosis.

Distinguishing these processes is important because they activate distinct signaling pathways, generate different biomarker profiles, and would respond differently to PAD4 inhibition or other targeted interventions. PAD4 inhibitors would effectively suppress LTH but not mitophagy-associated mtDNA release, whereas DNase therapy would degrade DNA-based structures but not arrest the underlying mitophagy defect.

## 4. NETosis in Innate and Adaptive Immunity

### 4.1. Antimicrobial Functions of NETs

Under homeostatic conditions and during acute infections, NETs contribute substantially to host defense through multiple mechanisms [12]. The physical mesh structure of NETs immobilizes diverse pathogens including bacteria, fungi, viruses, and parasites, preventing their dissemination and concentrating antimicrobial factors in high local concentrations [4]. This immobilization function operates independently of antibody opsonization or complement activation, representing an innate defense mechanism that functions in the absence of adaptive immunity.

The protein composition of NETs enables direct antimicrobial activity through multiple mechanisms. High concentrations of antimicrobial peptides, including LL37 and defensins, exert direct bactericidal and fungicidal effects; neutrophil elastase and other proteases degrade virulence factors; and myeloperoxidase catalyzes generation of hypochlorous acid, a potent antimicrobial oxidant [12]. Additionally, NETs restrict biofilm formation and can disrupt established biofilms, preventing the transition from planktonic to sessile bacterial growth that characterizes chronic infection. The capacity of NETs to disrupt established biofilms has led to investigation of NET-targeting strategies as adjunctive therapies for chronic bacterial infections associated with biofilm formation [12].

Pathogens have, however, evolved multiple mechanisms to evade NET-mediated killing. Bacterial nucleases can digest the extracellular DNA scaffold and facilitate escape; capsules and cell-surface charge modifications reduce trapping or confer resistance to cationic antimicrobial peptides; catalases, superoxide dismutases, and related stress-response systems blunt oxidative killing; and some pathogens suppress NET release or directly injure neutrophils through cytolysins and leukocidins. Biofilm formation provides an additional physical and biochemical barrier to NET penetration. These countermeasures emphasize that NETs are one component of host defense rather than an autonomous antimicrobial system [16].

### 4.2. NETs as Adjuvants of Adaptive Immunity

NETs exhibit important interconnections with adaptive immunity through multiple pathways. By concentrating and presenting antigens and pathogen-associated molecular patterns in high local concentrations, NETs facilitate antigen uptake and presentation by dendritic cells and other antigen-presenting cells, thereby promoting CD4+ T cell activation and B cell responses against pathogenic antigens [6]. Importantly, this adjuvant-like function of NETs supports adaptive immunity against infectious agents but becomes pathogenic in the context of autoimmunity where NET-displayed autoantigens (dsDNA, histones, nucleosomes) drive autoactive B cell and T cell responses.

The mechanisms by which NET-displayed autoantigens drive autoimmunity in SLE deserve particular emphasis. NETs expose conformationally modified versions of nuclear autoantigens that may bypass tolerance mechanisms; they concentrate autoantigens into immunological synapses, facilitating high-avidity interactions with autoreactive B cells; and they deliver autoantigens directly to B cell zones in secondary lymphoid tissues, promoting germinal center formation and production of high-affinity autoantibodies [17]. Furthermore, the combination of antigens (autoantigens) plus danger signals (NET-associated PAMPs/DAMPs) that NETs provide mimics the “two-signal” requirement for T cell and B cell activation, enabling autoreactive clones to become activated despite the presence of regulatory T cells.

### 4.3. Pathological Consequences of Dysregulated NETosis

When NETosis becomes dysregulated, the same properties that provide antimicrobial benefit become pathogenic [6]. Excessive NET formation releases large quantities of inflammatory mediators, proteases, and oxidants that directly injure tissues. The chromatin scaffold itself triggers innate immune receptors including TLRs, the inflammasome, and complement pathways. Autoantigens released from NETs drive the breakdown of self-tolerance and autoantibody production. Additionally, the persistence of NETs due to impaired clearance creates a chronic depot of immunostimulatory material that continuously activates innate immunity.

In SLE specifically, the convergence of excessive NET formation and impaired NET clearance creates a particularly problematic scenario in which NETs accumulate both in circulation and in affected tissues [5]. Tissue-deposited NETs and NET-associated immune complexes trigger local inflammation, complement activation, and recruitment of infiltrating immune cells, culminating in progressive tissue damage in multiple organs. The most severely affected organ, the kidney, develops lupus nephritis characterized by glomerular NET deposition, immune complex formation, and crescentic inflammation [18].

## 5. Molecular Mechanisms Linking NETs to Lupus Pathogenesis

### 5.1. Impaired NET Clearance and DNase Dysfunction

A fundamental mechanism predisposing patients with SLE to NET accumulation is impaired extracellular DNA clearance. DNase1 is a major circulating endonuclease, whereas DNase1L3 has distinct biochemical properties that enable digestion of chromatin and DNA associated with microparticles and other protein/lipid complexes. DNase1L3 is particularly linked to the immune system: human plasmacytoid dendritic cells show high DNASE1L3 expression, and monocytes, monocyte-derived dendritic cells, and macrophages can produce and secrete active DNase1L3 [19]. This immune-cell source distinguishes DNase1L3 biology from the predominantly nonimmune tissue sources of circulating DNase1 and helps explain why loss of DNase1L3 can directly connect defective disposal of extracellular chromatin with autoimmunity.

Rare biallelic loss-of-function variants in *DNASE1L3* cause a monogenic, frequently early-onset lupus phenotype and must be distinguished from the more common acquired or functional reduction of nuclease activity in sporadic SLE. In sporadic disease, neutralizing anti-DNase antibodies can contribute to impaired clearance. Anti-DNase1L3 antibodies were detected in approximately 30% of patients in one SLE cohort and were associated with greater disease activity, while reduced circulating DNase1L3 activity with neutralizing autoantibodies has been reported in more than half of patients with SLE nephritis in another study [20,21]. Earlier studies also reported anti-DNase I reactivity in substantial subsets of patients, although prevalence varies by assay and cohort. Thus, the term “DNase deficiency” in sporadic SLE is best understood as a heterogeneous state that may reflect reduced enzyme activity, inhibitory autoantibodies, altered secretion, or, rarely, inherited nuclease deficiency rather than a uniform genetic defect.

### 5.2. Excess NET Formation by Low-Density Granulocytes

While impaired NET clearance contributes to SLE pathogenesis, excess NET formation by low-density granulocytes (LDGs) represents an equally important mechanism [22]. LDGs are recovered in the peripheral blood mononuclear-cell fraction after density-gradient centrifugation and are expanded in many patients with active SLE. Rather than representing a single cell type, lupus LDGs are heterogeneous and include immature and intermediate/mature neutrophil populations with distinct transcriptional, epigenetic, and functional programs [23]. This heterogeneity is important when interpreting LDG abundance as a biomarker or assigning a single pathogenic function to the entire low-density compartment.

The mechanisms underlying LDG expansion remain incompletely understood. Current evidence suggests that altered granulopoiesis contributes to the increased abundance of LDGs in SLE. Inflammatory cytokines and myeloid growth factors, including G-CSF and GM-CSF, can promote emergency granulopoiesis, neutrophil mobilization, survival, and acquisition of activated phenotypes; in SLE, this growth-factor-rich inflammatory environment may favor release or persistence of low-density neutrophil populations. Genomic analyses support increased granulopoiesis as an important source of LDGs and show that LDG enrichment is associated with type I IFN- and TNF-responsive gene programs [23,24].

LDGs are also tightly linked to the type I interferon axis. Lupus LDGs display strong expression of interferon-stimulated genes and show altered responses to type I IFN, while NET-derived nucleic acids can activate plasmacytoid dendritic cells and other innate sensors to generate additional IFN-I. IFN-I in turn primes neutrophils for inflammatory activation and NET release, establishing a bidirectional LDG-NET-IFN amplification loop. Epigenetic studies provide a mechanistic basis for this phenotype: lupus neutrophils and autologous LDGs show demethylation of interferon-regulated genes, and chromatin-accessibility profiling identifies distinct epigenetic states among immature and mature LDG subsets [23,25]. These findings suggest that LDGs are not merely transiently activated cells but can be transcriptionally and epigenetically programmed toward interferon-responsive, vasculopathic, and NET-prone phenotypes.

### 5.3. LL37 and Antimicrobial Peptide-Mediated NET Activation

LL37 (cathelicidin), an antimicrobial peptide abundant in neutrophil granules and also produced by keratinocytes and epithelial cells, has emerged as an important amplifier of NET-mediated autoimmunity in SLE [5]. LL37 binds to self-DNA, and complexes of LL37 with self-DNA or RNA are potent activators of endosomal TLRs, particularly TLR7 and TLR9 [5]. In SLE skin tissue, elevated LL37 production by keratinocytes complexes with photodamaged DNA and NET-derived DNA to drive TLR7/9-dependent dendritic cell activation and production of Type I interferons, contributing to cutaneous lupus manifestations and systemic disease propagation.

Furthermore, LL37 itself has been identified as an autoantigen in SLE; circulating anti-LL37 antibodies are detected in a subset of SLE patients and correlate with disease activity [5]. The capacity of LL37 to form NET-like structures and to prime neutrophils for NETosis amplifies its pathogenic potential. These observations suggest that LL37-targeting strategies, potentially through sequestration or neutralizing antibodies, might represent an adjunctive approach to NETosis-targeted therapy.

### 5.4. HMGB1 and Danger-Associated Molecular Patterns

High-mobility group box 1 (HMGB1) protein, a nuclear and cytoplasmic protein abundant in neutrophils, functions as a critical danger-associated molecular pattern (DAMP) with potent immunostimulatory properties [26]. HMGB1 is released from dying cells, including those undergoing NETosis, and passively released HMGB1 in association with nucleosomes or DNA represents a particularly potent stimulus for dendritic cell activation and interferon production [27]. In SLE patients, circulating HMGB1 levels are elevated and correlate with disease activity, particularly in patients with lupus nephritis.

HMGB1 mediates its pro-inflammatory effects through multiple receptors, including the receptor for advanced glycation end products (RAGE) and TLR2/TLR4, triggering transcription factor activation and production of pro-inflammatory cytokines and interferons [26]. The combination of HMGB1 with NET-derived DNA or chromatin structures amplifies immunostimulation synergistically, as HMGB1 facilitates uptake of DNA and its delivery to endosomal TLRs. Recent evidence suggests that HMGB1-targeting strategies, including neutralizing antibodies or soluble receptors, might reduce NET-mediated inflammation and warrant investigation in SLE.

### 5.5. Mitochondrial DNA and the cGAS-STING Pathway

Accumulating evidence indicates that mitochondrial DNA (mtDNA), particularly oxidized mtDNA, is an important immunostimulatory component released by lupus neutrophils [28]. Ribonucleoprotein immune complexes and type I IFN priming promote mitochondrial ROS, oxidative modification of mitochondrial nucleoids, and extracellular release of interferogenic mtDNA. In patients with SLE, mtDNA-containing NETs and anti-mtDNA antibodies have been associated with disease activity and show a particularly strong relationship with lupus nephritis; mtDNA has also been demonstrated in NET-like structures in renal biopsy specimens [29]. These observations provide a clinically relevant link between abnormal mitochondrial handling in neutrophils and renal immune-complex inflammation.

Oxidized mtDNA can activate cytosolic DNA sensing through cyclic GMP-AMP synthase (cGAS), generating cGAMP and activating STING, TBK1, and IRF3 to promote type I interferon transcription. This pathway complements endosomal TLR9-dependent sensing of mitochondrial nucleic acids and provides a mechanism by which oxidized mtDNA may sustain systemic interferon signaling and organ inflammation. Pharmacologic STING antagonism is therefore an attractive strategy, but the translational evidence should be interpreted cautiously. Agents such as the experimental STING antagonist ISD017 have suppressed pathological cytokine responses in cells from patients with lupus and improved disease in lupus models, whereas definitive efficacy data from completed randomized clinical trials of STING inhibitors in SLE remain limited [30]. Thus, cGAS-STING inhibition remains an emerging rather than established clinical strategy.

### 5.6. Complement Activation and Immune Complex Formation

NETs promote activation of the complement cascade through multiple mechanisms, including exposure of complement-activating epitopes on histones and other NET-associated proteins, as well as sequestration of complement components within the NET structure [31]. In lupus nephritis specifically, tissue-deposited NETs activate the complement cascade directly and also serve as nucleation sites for immune complex formation through binding of circulating anti-dsDNA and anti-histone autoantibodies, forming pathogenic NET-immune complexes that induce further complement activation and glomerular injury.

Complement activation products including C3a and C5a amplify neutrophil recruitment and NETosis in a feed-forward manner, creating a vicious cycle in which NET-induced complement activation amplifies recruitment of additional neutrophils that undergo NETosis, increasing the NET burden. This feed-forward amplification contributes substantially to the rapid progression of lupus nephritis in patients experiencing flares. Therapeutic targeting of complement pathways downstream of NET deposition represents one rationale for investigating complement inhibitors in SLE.

### 5.7. Inflammasome Activation and IL-1β/IL-18 Amplification

The NLRP3 inflammasome, a molecular assembly comprising NLRP3 protein, apoptosis-associated speck-like protein (ASC), and pro-caspase-1, represents a critical converging point for multiple innate immune pathways dysregulated in SLE [32]. NET components, including DNA, histones, and oxidized lipids, activate the NLRP3 inflammasome through multiple mechanisms, leading to caspase-1-mediated cleavage of pro-IL-1β and pro-IL-18 into their active secreted forms. IL-1β and IL-18 are potent pro-inflammatory cytokines that amplify NETosis, create a proinflammatory milieu promoting disease progression, and contribute to both systemic manifestations and tissue-specific pathology.

Importantly, NET-induced inflammasome activation occurs in dendritic cells, macrophages, and other innate immune cells in response to NET uptake, creating a cell-autonomous amplification loop in which NETs → inflammasome activation → IL-1β production → further NETosis → accumulation of more NETs [32]. This cycle contributes substantially to disease progression in both active lupus nephritis and systemic manifestations. The identification of inflammasome activation as a critical checkpoint has prompted investigation of NLRP3 inhibitors and caspase-1 inhibitors as potential therapeutic approaches in SLE.

### 5.8. Endothelial Dysfunction and Vascular Inflammation

NETs and their components exert direct injurious effects on endothelial cells and vasculature through multiple mechanisms [33]. Neutrophil elastase and other proteases released from NETs degrade cell adhesion molecules and extracellular matrix proteins, compromising endothelial barrier function and increasing vascular permeability. Reactive oxygen species generated during NETosis and retained in NET structures cause endothelial oxidative stress and mitochondrial dysfunction. PAD4-mediated histone citrullination generates neoepitopes recognized by autoreactive antibodies, including anti-modified histone antibodies identified in SLE patients with cardiovascular disease.

In SLE patients, elevated NET markers predict the presence of subclinical atherosclerosis and coronary plaque burden, indicating that NET-mediated endothelial dysfunction contributes substantially to the accelerated atherosclerosis and cardiovascular disease characteristic of this population [34]. LDGs isolated from SLE patients demonstrate enhanced capacity to damage endothelial cells in vitro, and this endothelial damage correlates with elevated vascular inflammation markers. The contribution of NET-mediated endothelial dysfunction to SLE-associated cardiovascular complications represents an important clinical consideration in patient management.

### 5.9. Platelet Activation and Thrombotic Complications

NETs promote platelet activation and thrombosis through multiple mechanisms, including physical interaction between NET-associated tissue factor and platelet receptors, transfer of NET-associated phosphatidylserine to platelet surfaces, and NET-mediated complement activation on platelet surfaces [6]. Additionally, NETs bind to von Willebrand factor and collagen, promoting platelet adhesion and aggregation. The result of these processes is NET-driven thrombosis that contributes substantially to the vascular complications and hypercoagulability characteristic of SLE. Seminal work by Fuchs and colleagues established that extracellular DNA traps provide a physical and biochemical scaffold for thrombosis, supporting platelet adhesion and aggregation, recruiting red blood cells, and promoting fibrin deposition; dismantling the scaffold with DNase reduced thrombus formation [35].

SLE patients, particularly those with antiphospholipid antibodies, exhibit elevated circulating NET markers and increased risk of venous and arterial thrombosis [36]. In patients with SLE and concurrent antiphospholipid syndrome, the synergistic effects of antiphospholipid antibody-mediated tissue factor activation and NET-induced thrombosis create particularly high thrombotic risk. Some evidence suggests that NET-derived microparticles and phosphatidylserine-exposing structures contribute to the abnormal thrombin generation and prolonged clotting times observed in SLE with antiphospholipid syndrome.

### 5.10. Type I Interferon Signature and Systemic Disease Propagation

The Type I interferon signature—elevated expression of genes whose products are induced by Type I interferons (interferons-α/β)—represents one of the most consistent immunological hallmarks of SLE and serves as a biomarker of disease activity and predictor of organ involvement [1]. Multiple lines of evidence establish NETs and NET-associated nucleic acids as critical drivers of the Type I interferon signature in SLE. NETs release DNA and RNA that activate endosomal TLRs (TLR7, TLR9) and cytosolic sensors (cGAS-STING, RIG-I), collectively driving plasmacytoid dendritic cell production of Type I interferons [2].

The Type I interferon signal, in turn, perpetuates disease through multiple mechanisms: (1) priming neutrophils for enhanced NETosis in response to subsequent stimuli; (2) promoting B cell activation and autoantibody production; (3) driving T cell dysfunction and depletion of regulatory T cells; (4) enhancing migration of autoreactive cells to target organs; and (5) promoting endothelial dysfunction and tissue inflammation [2]. This feed-forward loop linking NETs → nucleic acid sensing → Type I interferon → enhanced NETosis represents a critical pathogenic axis in SLE. Breaking this cycle represents a major focus of emerging therapeutic strategies.

## 6. NETosis and Lupus Nephritis

### 6.1. Renal NET Deposition and Glomerular Pathology

Lupus nephritis represents the most common and clinically significant systemic manifestation of SLE, affecting 40–80% of patients and representing the leading cause of lupus-associated morbidity and mortality [18]. Compelling evidence implicates NETs in the pathogenesis of lupus nephritis through multiple mechanisms. Electron microscopy and immunofluorescence studies of renal biopsy tissue from lupus nephritis patients reveal extensive deposition of NETs within glomeruli, particularly in mesangial regions and at the glomerular basement membrane, with intense colocalization of NET components including MPO, neutrophil elastase, and citrullinated histones [18].

The mechanism of glomerular NET deposition involves several steps: (1) circulating NETs and NET remnants access the glomerular microvasculature due to increased vascular permeability during SLE flares; (2) NET-associated DNA and proteins interact with anti-dsDNA antibodies and other circulating autoantibodies, forming pathogenic immune complexes that deposit in glomeruli; (3) direct interactions between NET-associated tissue factor and thrombophilic components promote intravascular thrombosis within glomeruli; and (4) glomerular deposition of NETs activates the complement cascade locally, amplifying recruitment of infiltrating neutrophils that undergo additional NETosis [18].

Once deposited in the glomerulus, NETs perpetuate renal injury through multiple mechanisms. NET-associated proteases including neutrophil elastase and matrix metalloproteinases degrade structural proteins including collagen and extracellular matrix components, compromising glomerular architecture. NET-associated reactive oxygen species cause direct oxidative injury to podocytes, the specialized epithelial cells responsible for maintaining the glomerular filtration barrier [31]. PAD4-mediated histone citrullination generates neoepitopes that trigger local T cell and B cell responses. Most critically, NET deposition appears to drive the transition from early membranous/proliferative lupus nephritis to severe crescentic glomerulonephritis with extensive fibrinoid necrosis.

### 6.2. Podocyte Dysfunction and Proteinuria

Recent evidence indicates that NETs exert direct injurious effects on podocytes, the specialized epithelial cells responsible for the selectivity and permeability properties of the glomerular filtration barrier [31]. Podocytes exposed to NETs or NET-derived components in vitro exhibit increased permeability, disrupted cytoskeletal organization, and activation of apoptotic pathways. In lupus nephritis patients, the degree of glomerular podocyte loss correlates with NET marker levels and predicts progression to end-stage renal disease.

The mechanisms of NET-mediated podocyte injury include: (1) direct toxicity from neutrophil elastase and other proteases, which degrade podocyte junction proteins; (2) oxidative stress from NET-associated ROS; (3) complement activation cascades triggered by NET deposition; and (4) activation of pattern recognition receptors on podocytes by NET-associated PAMPs/DAMPs. The result is proteinuria through increased glomerular permeability and progressive podocyte depletion through apoptosis, eventually culminating in glomerulosclerosis and renal function loss.

### 6.3. Tubulointerstitial Involvement

While most attention has focused on glomerular pathology in lupus nephritis, tubular and interstitial compartments also exhibit NET deposition and NET-mediated injury [31]. NET accumulation in the interstitium promotes infiltration of inflammatory immune cells, interstitial inflammation, and activation of fibrotic pathways. Neutrophil elastase and other NET-associated proteases may contribute to tubular basement membrane disruption and tubular epithelial cell injury.

The tubulointerstitial component of lupus nephritis appears particularly important for predicting progression to end-stage renal disease and represents an area where NET-targeted therapies might provide particular benefit. Indeed, preclinical studies demonstrate that reducing NETosis or degrading accumulated NETs ameliorates both glomerular and tubulointerstitial pathology in lupus nephritis models.

## 7. NETosis and Neuropsychiatric Lupus

### 7.1. Central Nervous System Involvement in SLE

Neuropsychiatric manifestations affect 20–75% of SLE patients depending on disease duration and severity, and represent a major source of morbidity [37]. These manifestations encompass diverse syndromes including cognitive dysfunction, psychosis, mood disorders, seizures, cerebral vasculitis, stroke, and transverse myelitis. Transverse myelitis is a recognized inflammatory NPSLE syndrome that can cause acute or subacute motor, sensory, and autonomic deficits and requires prompt evaluation. While the pathogenesis of neuropsychiatric SLE (NPSLE) remains incompletely understood, emerging evidence implicates neutrophils and NETs in disease pathogenesis [37].

### 7.2. Blood–Brain Barrier Disruption

A critical mechanism whereby NETs potentially contribute to NPSLE involves disruption of the blood–brain barrier (BBB), the specialized endothelial barrier that maintains CNS immune privilege [37]. Neutrophils and NETs accumulate in perivascular regions surrounding cerebral vessels in NPSLE patients, and NET-associated proteases including neutrophil elastase directly degrade tight junction proteins that maintain BBB integrity. Complement activation triggered by NETs damages cerebral endothelial cells. ROS produced during NETosis and retained in NET structures cause endothelial oxidative injury.

The consequence is BBB breakdown, enabling infiltration of autoimmune cells and circulating autoantibodies into the CNS, where they encounter autoantigens including neuronal surface antigens and directly trigger neuroinflammation [37]. This BBB disruption appears particularly prominent in NPSLE patients with acute cognitive dysfunction and psychosis, and correlates with elevated CSF inflammatory markers and imaging evidence of CNS inflammation. The major downstream immune, vascular, and organ-specific consequences of persistent NETs in SLE are summarized in Figure 2.

### 7.3. Neuronal Autoimmunity

NETs expose neuronal autoantigens including NMDA receptor epitopes that trigger anti-NMDA antibody production in SLE patients, particularly those with NPSLE manifestations [37]. These anti-NMDA antibodies, in combination with anti-DNA antibodies and other circulating autoantibodies, penetrate the disrupted BBB and directly engage neuronal surface receptors, triggering internalization, receptor cross-linking, and subsequent neuronal injury through multiple pathways including calcium dysregulation and caspase activation.

Additionally, NETs stimulate microglia and astrocytes, CNS resident immune cells, to produce pro-inflammatory cytokines and complement factors that directly injure neurons and oligodendrocytes. The combination of BBB disruption, circulating autoantibody infiltration, and local CNS innate immune activation creates a hostile neuroinflammatory microenvironment that explains the progressive cognitive and neuropsychiatric dysfunction characteristic of active NPSLE.

### 7.4. Potential Therapeutic Implications

The identification of NETosis and NETs as contributors to NPSLE pathogenesis suggests that NET-targeted therapies might ameliorate cognitive dysfunction and neuropsychiatric manifestations in SLE. Initial preclinical studies indicate that DNase therapy or PAD4 inhibition reduces neuroinflammation and cognitive dysfunction in lupus-prone mice with NPSLE-like manifestations. These observations warrant investigation in SLE patients presenting with active neuropsychiatric disease, particularly those with evidence of BBB disruption or elevated CSF inflammatory markers. Longitudinal studies correlating NET burden with cognitive decline and neuroimaging evidence of CNS disease activity will be essential to establish whether NET-targeted interventions can prevent or reverse NPSLE progression.

## 8. NETosis and Cardiovascular Complications in SLE

### 8.1. Accelerated Atherosclerosis and Arterial Injury

Patients with SLE experience dramatically accelerated atherosclerosis and premature cardiovascular disease, with cardiovascular events occurring 5–10 times more frequently than in age- and sex-matched controls [38]. While traditional risk factors including hypertension, dyslipidemia, and obesity contribute to this excess cardiovascular morbidity, they account for only a portion of the observed risk, suggesting that SLE-specific immunopathological mechanisms drive accelerated vascular disease. Emerging evidence implicates NETosis and NETs as central mediators of SLE-associated atherosclerotic progression and arterial injury [39].

NETs accumulate within atherosclerotic plaques and promote foam cell formation through NET-mediated uptake of modified lipoproteins by macrophages, a process facilitated by NET-associated phosphatidylserine and high concentrations of neutrophil proteases [38]. Additionally, circulating immune complexes containing anti-DNA antibodies and NET-derived autoantigens directly activate endothelial cells and promote atherosclerotic plaque destabilization through increased expression of adhesion molecules, tissue factor, and matrix metalloproteinases. The result is enhanced vascular inflammation, plaque erosion, and increased thrombotic potential, contributing to the elevated rates of acute myocardial infarction and stroke in SLE. Traditional risk factors are not necessarily low in this population: hypertension, dyslipidemia, obesity, insulin resistance, and glucocorticoid-associated metabolic effects can coexist with accelerated vascular aging. The excess cardiovascular risk therefore reflects the combined effects of conventional risk factors and SLE-specific mechanisms, including chronic inflammation, autoantibodies, endothelial dysfunction, complement activation, and NET-mediated vascular injury.

### 8.2. Endothelial Dysfunction and Vasculitis

NETs directly injure vascular endothelial cells through multiple mechanisms [38]. NET-associated proteases, including neutrophil elastase and matrix metalloproteinase-9, degrade endothelial glycocalyx and extracellular matrix, disrupting the endothelial barrier and promoting vascular leak. Simultaneously, NET-derived histones and HMGB1 bind to endothelial TLRs and death receptors, triggering endothelial apoptosis, enhanced expression of pro-coagulant tissue factor, and production of prothrombotic microparticles. These endothelial perturbations explain the vasculitis observed in cutaneous and systemic SLE manifestations and contribute to the pathogenesis of lupus vasculitis affecting medium and large vessels.

Anti-endothelial cell antibodies present in SLE patients’ sera cross-react with NET-associated antigens, amplifying endothelial injury through complement-dependent and antibody-dependent cellular cytotoxicity mechanisms. The combination of direct NET-mediated endothelial injury, autoantibody-mediated complement activation, and tissue factor-driven local coagulation creates microenvironments of intense vascular inflammation and thrombosis that characterize lupus vasculitis and contribute substantially to SLE-associated cardiovascular morbidity and mortality.

### 8.3. Thrombosis and Antiphospholipid Syndrome

A subset of SLE patients develop antiphospholipid syndrome (APS), characterized by thrombosis and pregnancy morbidity associated with circulating antiphospholipid antibodies. Emerging evidence indicates that NETosis links innate immune activation to the thrombotic phenotype of APS [38]. NETs expose tissue factor and phosphatidylserine on their scaffolds, providing a potent pro-coagulant surface that drives thrombin generation and platelet activation. Furthermore, NET-bound tissue factor preferentially activates protease-activated receptor-2 on platelets and endothelial cells, promoting platelet aggregation and thrombotic microparticle release.

Antiphospholipid antibodies directly activate neutrophils through TLR7 and TLR8, enhancing NETosis and NET-mediated tissue factor exposure. This creates a feed-forward cycle where autoimmune activation drives enhanced NETosis, which in turn exposes pro-thrombotic NET-associated antigens that trigger further autoantibody production and complement activation. This mechanistic link between NETosis and thrombosis suggests that NET-targeted therapies might prevent thrombotic complications in APS patients and reduce the alarmingly high rates of recurrent thrombotic events in this high-risk SLE population.

## 9. NETosis as a Diagnostic and Prognostic Biomarker

### 9.1. Circulating NETosis Biomarkers

The measurement of circulating biomarkers reflecting NETosis has emerged as a promising approach for disease monitoring, prognostication, and patient stratification in SLE [11]. Cell-free DNA (cfDNA), particularly circulating neutrophil-derived nuclear DNA, serves as a non-specific but readily measurable marker of NETosis and correlates with SLE disease activity, lupus nephritis severity, and organ damage accumulation. Elevated cfDNA levels associate with elevated anti-DNA antibody titers and complement consumption, suggesting that circulating NET components directly contribute to immune complex formation and renal pathology.

More specific NETosis biomarkers include myeloperoxidase-DNA (MPO-DNA) complexes, which reflect association of neutrophil granule MPO with extracellular DNA, and citrullinated histone H3 (H3cit), a product of PAD4-mediated histone modification during NET formation [11]. These markers show dramatically elevated concentrations in SLE patients compared to healthy controls and other rheumatic diseases including rheumatoid arthritis, suggesting disease-specific alterations in NETosis regulation in SLE. Importantly, patients with active lupus nephritis or cardiovascular complications display significantly elevated NET-associated biomarker levels, indicating that these biomarkers may identify patients at highest risk for severe organ manifestations. The combination of multiple NETosis biomarkers, including cfDNA, MPO-DNA complexes, H3cit, and circulating nucleosomes, provides complementary information and may enable more accurate risk stratification than any single marker alone [11]. Key NETosis biomarkers and their potential clinical applications are summarized in Figure 3.

Calprotectin (S100A8/A9), one of the most abundant cytosolic protein complexes in neutrophils, is another relevant neutrophil-associated biomarker in SLE. S100A8/A9 can be released during neutrophil activation and is present in inflammatory extracellular material, including NET-rich environments. Circulating S100A8/A9 concentrations are elevated in SLE and have been associated with organ damage and cardiovascular disease [40]. However, calprotectin is not specific for NETosis because it is also released by activated neutrophils and monocytes independently of NET formation. It is therefore best interpreted as a marker of myeloid inflammation that can complement, rather than replace, more structurally NET-associated measurements such as MPO-DNA or neutrophil elastase-DNA complexes.

### 9.2. NETosis Biomarkers and Disease Activity Monitoring

Longitudinal studies demonstrate that circulating NETosis biomarker levels track with SLE disease activity, rising during lupus flares and declining during remission [41]. Platelet-associated NETosis parameters, measured through specialized flow cytometry assays quantifying early and late stages of NET formation in mixed cultures of neutrophils and platelets, show particular promise as informative laboratory markers of inflammatory activity. During acute disease exacerbations, platelet-associated NETosis parameters increase substantially compared to baseline, and these parameters decline toward control values when clinical remission is achieved through intensified immunosuppressive therapy [41].

The LDG-to-lymphocyte ratio, incorporating the elevated numbers of LDGs present during active SLE, correlates more strongly with SLE disease activity index (SLEDAI) scores than traditional neutrophil-to-lymphocyte ratios, suggesting that quantifying disease-specific neutrophil subsets improves disease monitoring [8]. These findings suggest that incorporating NETosis biomarker measurement into routine SLE clinic practice could enable more objective assessment of disease activity, guide intensification or de-escalation of immunosuppressive therapy, and potentially enable early detection of impending flares before clinical manifestations emerge.

### 9.3. Prognostic Stratification and Organ Involvement Prediction

Emerging evidence indicates that baseline NETosis biomarker levels predict future organ involvement and disease progression [38]. SLE patients with elevated baseline cfDNA, MPO, and H3cit levels show higher incidence of lupus nephritis development over subsequent follow-up, even among patients without renal disease at baseline. Similarly, elevated circulating NETosis biomarkers associate with increased rates of cardiovascular events, thrombosis, and neuropsychiatric manifestations during longitudinal follow-up, suggesting prognostic utility for disease stratification and risk-adapted treatment intensification.

The association between LDG proportions and shortened pregnancy duration in pregnant women with SLE highlights the potential for NETosis biomarkers to predict pregnancy complications and guide antenatal monitoring [42]. These observations collectively suggest that comprehensive immune profiling incorporating NETosis biomarkers, combined with clinical and serological assessment, could enable precision medicine approaches that identify high-risk patient subsets amenable to preventive or intensified interventions. Future prospective studies should establish the optimal timing, frequency, and combination of NETosis biomarker measurements for clinical practice and validate whether biomarker-guided therapy intensification improves long-term outcomes.

## 10. Therapeutic Approaches Targeting NETosis

### 10.1. PAD4 Inhibitors and NET Formation Inhibition

Peptidylarginine deiminase 4 (PAD4) catalyzes histone citrullination and can facilitate chromatin decondensation in selected NET-forming pathways [14]. PAD4 inhibition therefore remains an attractive strategy for reducing citrullination-associated NET formation. However, NET release is mechanistically heterogeneous and not universally PAD4-dependent. Vital NET release and other stimulus-specific pathways may proceed with little requirement for PAD4, and genetic studies have produced model-dependent results. In MRL.Faslpr mice, *Padi4* deletion failed to improve nephritis, autoantibody formation, or immune activation [15], whereas other inflammatory lupus models have shown benefit from PAD4 deficiency or inhibition. These findings help explain why broad PAD4 blockade cannot be assumed to suppress all pathogenic extracellular chromatin release.

Accordingly, future PAD4-directed therapies should be paired with biomarkers demonstrating PAD4-dependent citrullination rather than selected solely on the presence of circulating extracellular DNA. Neutrophil-targeted delivery systems may improve cellular exposure and limit off-target effects [43], but clinical translation will require demonstration that the relevant SLE endotype is genuinely driven by PAD4-dependent NET formation. The mixed preclinical literature also underscores the importance of preserving antimicrobial neutrophil functions and avoiding overinterpretation of reductions in citrullinated histones as evidence that all NET pathways have been inhibited.

### 10.2. DNase Therapy and NET Clearance Enhancement

DNase therapy is one of the most direct approaches to reducing extracellular chromatin burden because it dismantles the DNA scaffold of existing NETs rather than preventing their formation. Early clinical experience with recombinant human DNase I (rhDNase) illustrates both the rationale and the pharmacologic challenges. In a randomized, double-masked phase Ib study of 17 patients with lupus nephritis, intravenous rhDNase was well tolerated and transiently produced serum hydrolytic activity, but subcutaneous administration did not achieve measurable hydrolytic activity and short-term treatment did not improve anti-dsDNA antibodies, complement, circulating immune complexes, or other disease-activity markers [44]. The estimated serum half-life after intravenous dosing was only several hours, highlighting the limitations of stability, systemic exposure, and bioavailability.

A recent first-in-human proof-of-concept report described therapeutic administration of a recombinant human DNASE1L3 analogue in a patient with severe, treatment-refractory SLE caused by congenital *DNASE1L3* deficiency [45]. Recombinant DNASE1L3 was administered intravenously at 3 mg/kg once weekly, resulting in supraphysiologic plasma DNASE1L3 levels and a strikingly rapid clinical response. Within 6 h after the first infusion, marked improvement or resolution of longstanding manifestations, including episcleritis, arthritis, vasculitic rash, and fatigue, was observed. This clinical improvement coincided with a rapid increase in circulating DNA and MPO–DNA complexes, consistent with enzymatic degradation of NETs in tissues. Although this observation is limited to a single patient with congenital DNASE1L3 deficiency, it provides first-in-human proof of concept that therapeutic degradation of NETs can rapidly ameliorate severe SLE manifestations and supports further investigation of NET-targeted nuclease therapy [45].

Several barriers remain. Exogenous nucleases must retain activity in plasma and tissues, reach sites of NET deposition, and avoid rapid clearance or neutralization. Pre-existing anti-DNase1 or anti-DNase1L3 autoantibodies may inhibit endogenous or administered enzymes in some patients [20,21], although neutralizing antibodies to rhDNase were not detected in the short 1999 trial [44]. Moreover, DNA cleavage does not necessarily neutralize histones, proteases, tissue factor, or other toxic NET-associated proteins and could generate smaller nucleoprotein fragments with residual immunostimulatory activity. These considerations favor engineered nucleases with improved pharmacokinetics, combination strategies that neutralize NET proteins, and selection of patients with demonstrable nuclease insufficiency.

### 10.3. JAK Inhibitors and Type I Interferon Pathway Suppression

JAK inhibitors exert multitargeted immune suppression by blocking signaling of multiple cytokines including type I interferons, which are crucial drivers of NETosis in SLE [46]. These agents reduce interferon-α production and restore immune homeostasis through multiple mechanisms including inhibition of STAT1 and STAT3 signaling, downstream targets of JAK-mediated cytokine signaling. Importantly, JAK inhibition broadly suppresses type I interferon signature genes that are characteristically elevated in SLE patient neutrophils and immune cells. This elevated interferon signature predisposes neutrophils toward enhanced spontaneous NETosis and contributes substantially to the pro-inflammatory phenotype of SLE immune cells.

JAK inhibitors currently under investigation for SLE include baricitinib, upadacitinib, and filgotinib, with several phase 2b and phase 3 clinical trials demonstrating clinical efficacy in both cutaneous and systemic SLE manifestations [46]. The immunomodulatory effects of JAK inhibitors likely operate through multiple mechanisms including enhanced regulatory T cell differentiation, reduced Th17 cell development, and diminished B cell activation, in addition to their effects on NETosis regulation. However, important safety concerns persist—including infections, particularly herpes zoster, and thromboembolism in SLE patients with concomitant antiphospholipid syndrome—necessitating careful patient selection and monitoring during JAK inhibitor therapy.

### 10.4. Complement Inhibitors and NET-Mediated Complement Activation

Complement inhibition is particularly relevant to lupus nephritis because NETs and immune complexes can amplify both classical and alternative pathway activation, while complement-derived anaphylatoxins further recruit and activate neutrophils. Clinical development has therefore moved beyond terminal C5 blockade toward proximal pathway targets. Iptacopan (LNP023), an oral factor B inhibitor, is being evaluated in a randomized phase 2 trial in active class III/IV ± V lupus nephritis (NCT05268289) [47]. Pegcetacoplan (APL-2), which binds C3 and prevents generation of C3a and C3b, has been evaluated in a phase 2 study that included patients with lupus nephritis among several glomerulopathies (NCT03453619) [48].

The classical pathway is also being targeted more proximally. ANX009, a subcutaneously administered antigen-binding fragment directed against C1q, completed a small phase 1b study in active lupus nephritis (NCT05780515), providing early evidence of target engagement and complement-pathway inhibition, although efficacy conclusions remain preliminary [49]. These agents are not NET-specific therapies; their relevance to NET biology lies in interrupting NET-complement feed-forward amplification. Future studies should determine whether markers of NET burden or complement activation identify patients most likely to benefit and should carefully monitor infection risk associated with proximal complement blockade.

### 10.5. Oxidative Stress-Targeting Antioxidant Therapies

Oxidative stress is mechanistically linked to several forms of NET generation, but antioxidant therapy in SLE should be considered pathway-specific rather than as a single therapeutic class. N-acetylcysteine (NAC) replenishes intracellular glutathione and modifies redox-sensitive signaling; in a randomized double-blind placebo-controlled pilot trial, NAC reduced SLEDAI and BILAG disease-activity scores and inhibited mTOR signaling in T cells [50]. Mitochondria-targeted antioxidants provide a more direct approach to mitochondrial ROS: in MRL-lpr mice, MitoQ reduced neutrophil ROS, NET formation, type I interferon signaling, renal immune-complex deposition, and kidney disease [51]. Other agents, including curcumin and resveratrol, have antioxidant and anti-inflammatory effects and have shown signals of benefit in experimental or small clinical studies, but their effects cannot be attributed specifically to NET inhibition.

Important limitations include poor or variable bioavailability of many antioxidant compounds, uncertain tissue concentrations, pleiotropic mechanisms, dose-dependent effects, and the physiologic requirement for ROS in antimicrobial killing and cell signaling. Global ROS suppression could therefore impair host defense without adequately targeting the pathogenic ROS compartment. Future studies should prioritize pharmacologically defined agents, mitochondrial or NADPH-oxidase pathway specificity, validated oxidative-stress and NET biomarkers, and adequately powered clinical trials that distinguish general anti-inflammatory effects from direct modulation of NETosis.

## 11. Conclusions and Future Directions

The field of NETosis research has undergone significant evolution over the past decade, with fundamental reappraisals of its role in SLE pathogenesis leading to more nuanced understanding of how aberrant neutrophil function contributes to autoimmune activation and tissue injury. NETosis represents a complex and multifaceted process that operates at the intersection of innate immunity, autoimmunity, and inflammation in SLE. The identification and characterization of low-density granulocytes as specialized NET-forming cells has provided important insights into neutrophil heterogeneity and revealed mechanistic links between these cells and SLE severity, disease activity, and organ complications.

The role of PAD4 and protein citrullination in NET formation requires continued investigation to clarify the distinction between true NETosis and morphologically similar processes such as leukotoxic hypercitrullination and defective mitophagy. The measurement of circulating NETosis biomarkers, including cell-free DNA, myeloperoxidase, and citrullinated histones, has demonstrated promise as tools for disease monitoring and patient stratification. The connection between NETosis, the interferon signature, and autoantibody production highlights the need for systems-level approaches to understanding SLE pathogenesis that integrate multiple immunological abnormalities.

Future therapeutic efforts should build upon emerging understanding of NETosis mechanisms to develop targeted interventions that specifically inhibit aberrant NET formation while preserving the antimicrobial functions of these structures. Precision medicine approaches that employ comprehensive immune profiling and biomarker measurement may enable identification of patient subsets most likely to benefit from NETosis-targeting therapies. Additionally, investigation of oxidative stress-targeting approaches offers complementary therapeutic strategies addressing fundamental mechanisms of SLE pathogenesis. Ultimately, the convergence of mechanistic understanding, biomarker development, and innovative therapeutic approaches targeting multiple dysregulated pathways holds promise for fundamentally improving outcomes in SLE and related autoimmune diseases.

## Figures and Tables

**Figure 1:**
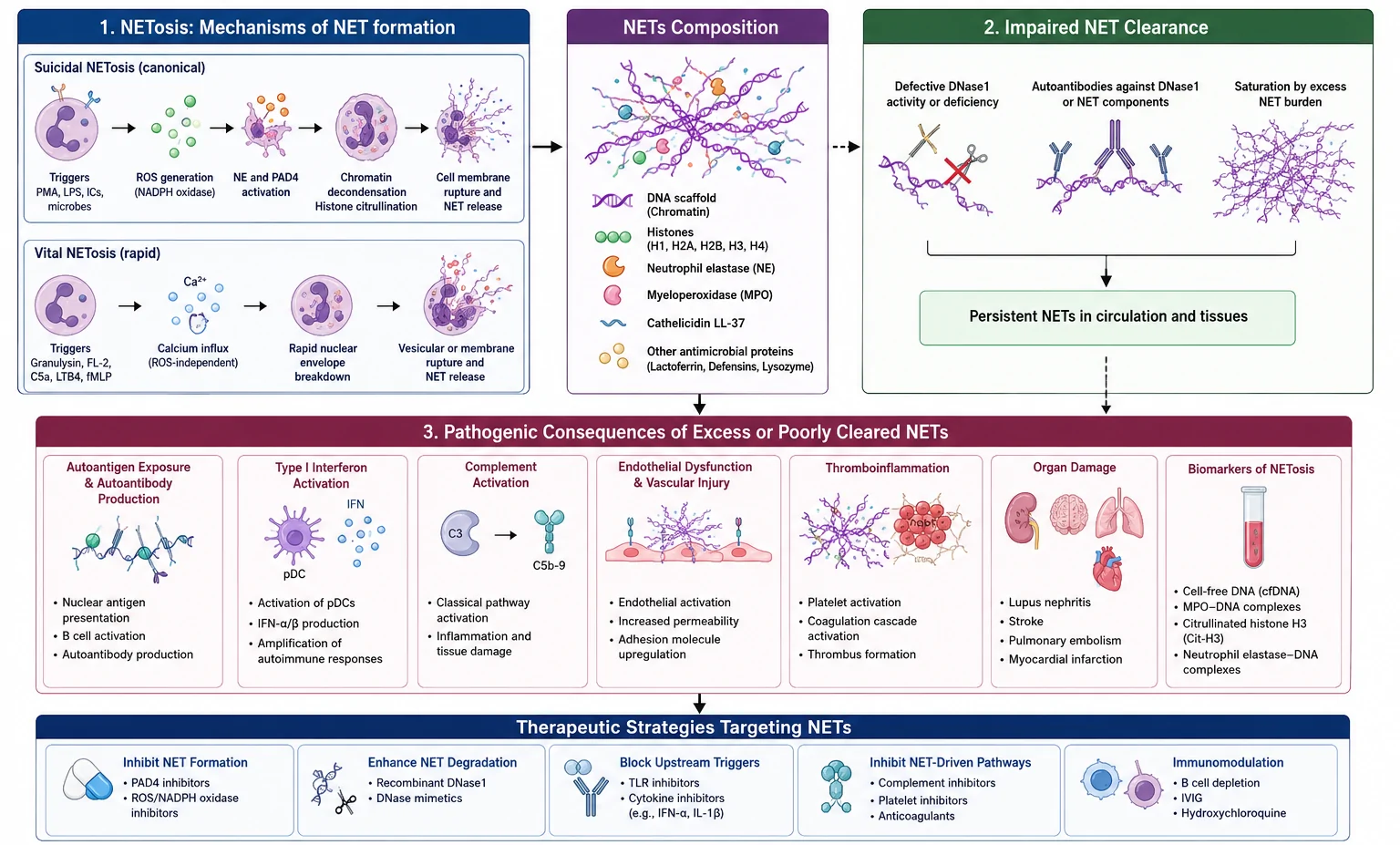
Mechanisms of neutrophil extracellular trap (NET) formation (NETosis). Schematic illustration of the two major pathways of NET formation. Canonical (suicidal) NETosis is initiated by stimuli such as phorbol 12-myristate 13-acetate (PMA), lipopolysaccharide (LPS), immune complexes (ICs), and microorganisms, leading to NADPH oxidase-dependent reactive oxygen species (ROS) generation, activation of neutrophil elastase (NE) and peptidylarginine deiminase 4 (PAD4), chromatin decondensation, histone citrullination, nuclear membrane disintegration, and eventual plasma membrane rupture with NET release. In contrast, vital NETosis is rapidly induced by stimuli including granulysin, FL-2, C5a, LTB4, and fMLP through ROS-independent calcium influx, resulting in rapid nuclear envelope breakdown and NET release while preserving neutrophil viability in certain settings. NETs consist of decondensed chromatin decorated with histones, myeloperoxidase (MPO), neutrophil elastase, LL-37, and other antimicrobial proteins. Although NETs contribute to pathogen capture and antimicrobial defense, excessive or dysregulated NETosis promotes autoantigen exposure, type I interferon signaling, complement activation, endothelial dysfunction, thrombosis, and tissue injury. Solid arrows indicate the direction or progression of the depicted biological processes, whereas dashed arrows indicate indirect or proposed mechanistic relationships between major processes. The scissors/nuclease symbol represents DNase1-mediated cleavage of NET DNA, and the red cross (×) indicates defective or impaired DNase1-mediated NET degradation.

**Figure 2:**
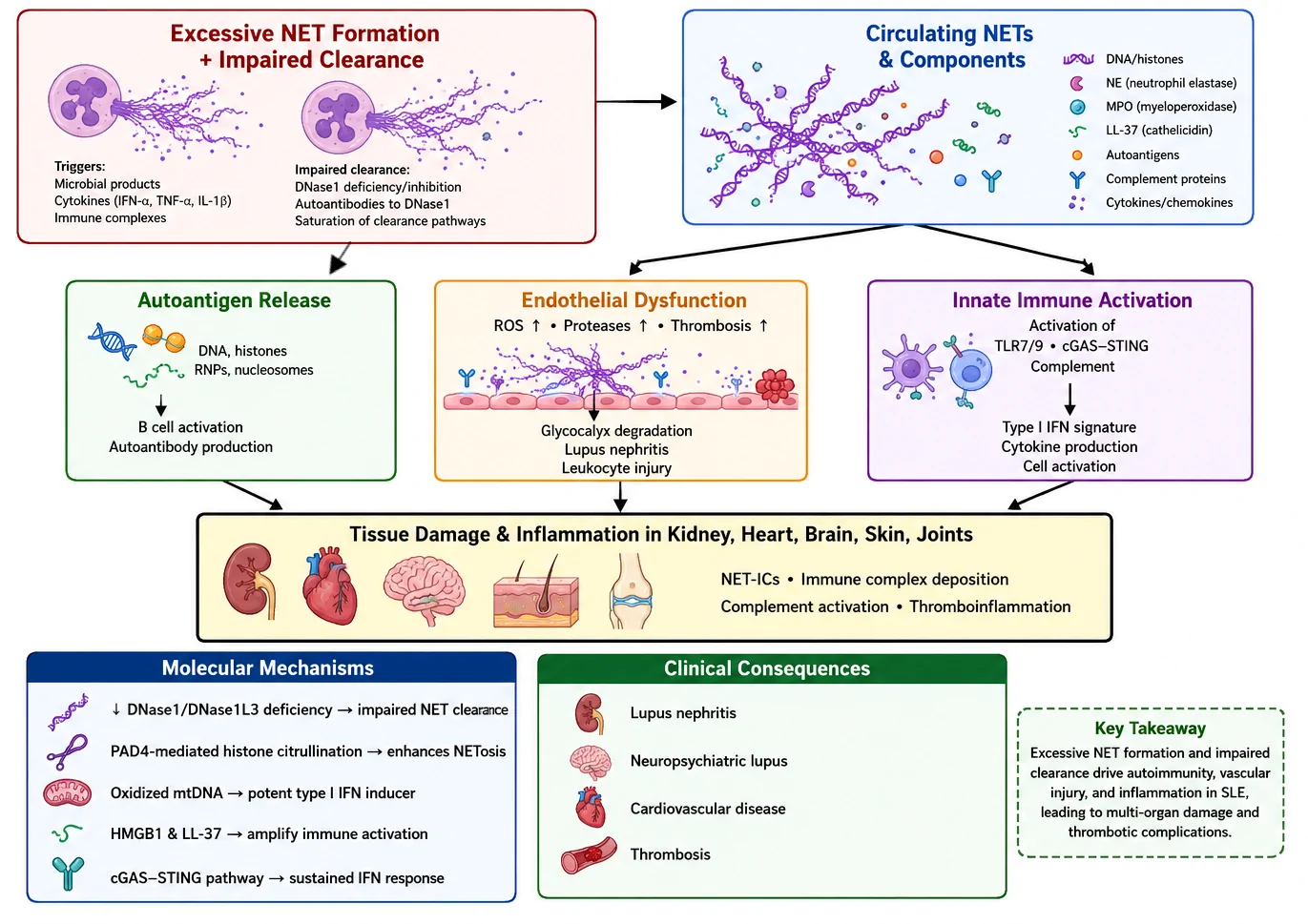
Integrated downstream consequences of persistent NETs in systemic lupus erythematosus (SLE). This schematic illustrates how persistent circulating NETs connect autoantigen exposure with B-cell autoimmunity, endothelial dysfunction and thromboinflammation, nucleic-acid sensing and type I interferon signaling, complement activation, and organ-specific injury. These convergent pathways contribute to lupus nephritis, neuropsychiatric lupus, cardiovascular disease, cutaneous and joint inflammation, and thrombosis. Upward (↑) and downward (↓) arrows indicate increased and decreased levels or activity, respectively, whereas connecting arrows indicate the proposed direction of mechanistic relationships.

**Figure 3:**
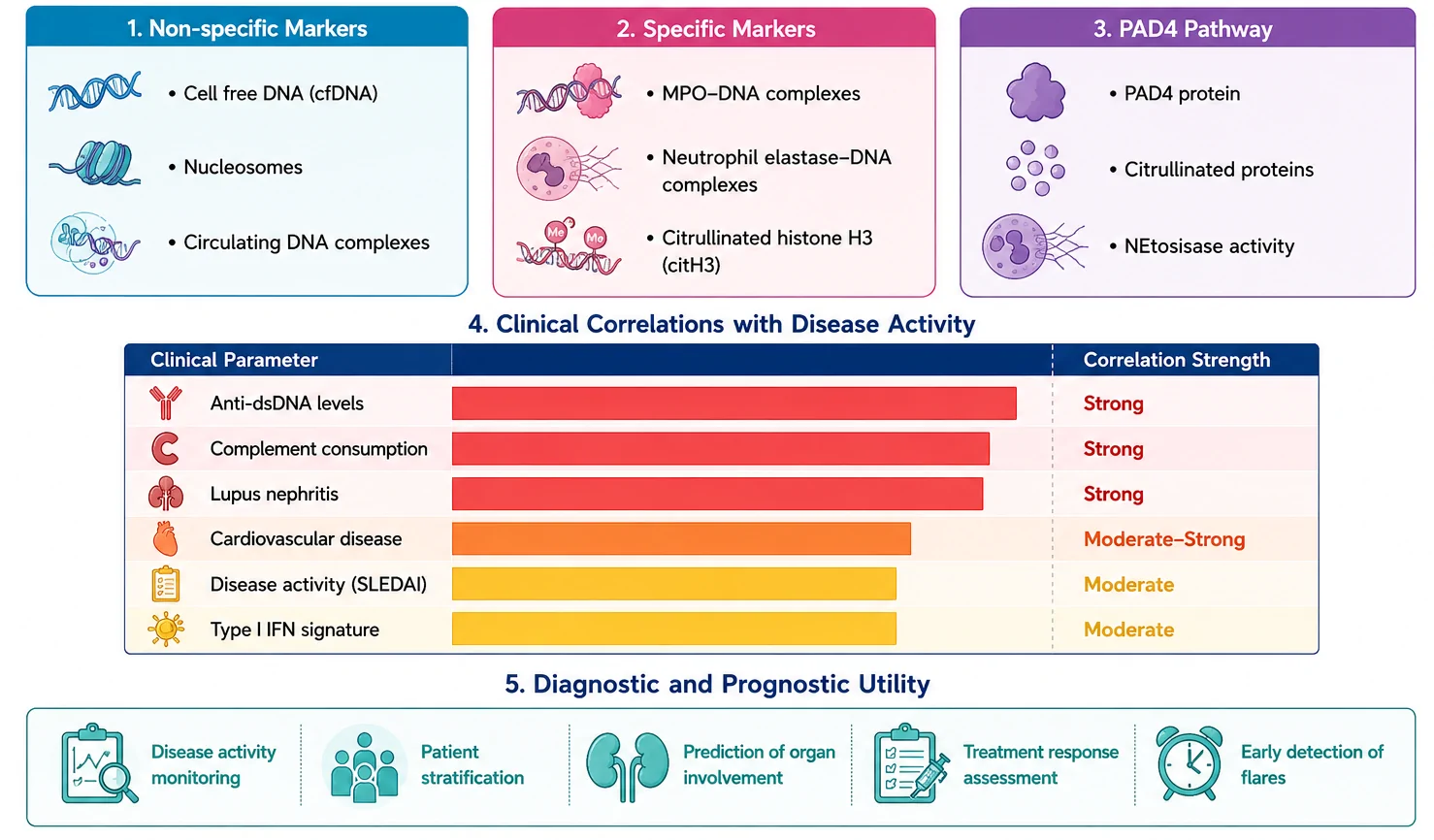
NETosis-associated biomarkers and their clinical significance in systemic lupus erythematosus (SLE). Biomarkers of NETosis include nonspecific markers such as cell-free DNA (cfDNA), nucleosomes, and circulating DNA complexes, as well as more NET-specific biomarkers including MPO–DNA complexes, neutrophil elastase–DNA complexes, and citrullinated histone H3 (Cit-H3). Biomarkers reflecting PAD4 activation, including PAD4 protein expression, citrullinated proteins, and neutrophil elastase activity, provide additional evidence of NET formation. Increased NET biomarkers correlate with disease activity, anti-double-stranded DNA (anti-dsDNA) antibody titers, complement consumption, lupus nephritis, cardiovascular complications, and type I interferon signatures. These biomarkers have potential applications in disease activity monitoring, patient stratification, prediction of organ involvement, treatment response assessment, and early detection of disease flares. However, their routine clinical implementation remains limited by the lack of standardized assays, variability among patient populations, and insufficient prospective validation.

## Data Availability

No new data were created or analyzed in this study. Data sharing is not applicable to this article.
